# Assessment of Antifungal Activity of Bakuchiol on Oral-Associated *Candida* spp.

**DOI:** 10.1155/2015/918624

**Published:** 2015-11-08

**Authors:** Mohd-Al-Faisal Nordin, Fathilah Abdul Razak, Wan Harun Himratul-Aznita

**Affiliations:** Department of Oral Biology and Biomedical Sciences, Faculty of Dentistry, University of Malaya, 50603 Kuala Lumpur, Malaysia

## Abstract

Bakuchiol is an active component of* Psoralea glandulosa* and* Psoralea corylifolia*, used in traditional Chinese medicine. The study aimed at investigating the antifungal activity of bakuchiol on planktonic and biofilm forms of orally associated* Candida* species. The antifungal susceptibility testing was determined by the broth micro dilution technique. Growth kinetics and cell surface hydrophobicity (CSH) of* Candida* were measured to assess the inhibitory effect of bakuchiol on* Candida* planktonic cells. Biofilm biomass and cellular metabolic activity were quantitatively estimated by the crystal violet (CV) and the 2,3-bis(2-methoxy-4-nitro-5-sulfophenyl)-5-[(phenylamino)carbonyl]-2H-tetrazolium hydroxide (XTT) assays. All* Candida* strains have been shown to be susceptible to bakuchiol with the MIC ranges from 12.5 to 100 *μ*g/mL. Significant decrease in specific growth rates and viable counts demonstrates the inhibitory effect of bakuchiol on* Candida* planktonic cells. A brief exposure to bakuchiol also reduced CSH of* Candida* (*P* < 0.05), indicating altered surface properties of yeast cells towards hydrophobic interfaces. Biofilm biomass and cell metabolic activity were mostly decreased, except for* C. glabrata* (*P* = 0.29). The antifungal properties of bakuchiol on* Candida* species in this* in vitro* study may give insights into the application in therapeutic strategy against* Candida* infections.

## 1. Introduction

Given the fact that at least 50% of healthy individuals are the carriers of* Candida* species, the commensal organisms are regarded as potentially pathogenic in susceptible hosts [1]. The infection is primarily depending on the immunological status of the host. Both local and systemic risk factors may result in weakened immune functions that mediate* Candida* colonisation on host surfaces [2].* Candida* species has a vibrant cell surface embedded with protein components that favour physical interaction to host surfaces. The adherence mechanisms are possibly mediated through both nonspecific and specific bindings. Previous studies reported that the cell surface hydrophobicity (CSH) of* Candida* yeast cells is a putative virulence factor, and its expressed proteins may affect the CSH status of* Candida* to interact with the cells and the surfaces [3, 4]. In addition, salivary pellicle components such as statherin and *α*-amylase and the complex bindings through lectin-like or protein-protein-type interactions have been known to be responsible for cell colonisation in disease progression [5, 6].

The pathogenesis of oral candidiasis has been closely associated with the biofilm formation of* Candida* on the oral surfaces [7]. Biofilm displays distinct biological properties compared to its planktonic counterparts which enable resistance to antifungal drugs.* C. albicans* biofilm consists of polysaccharide matrix-enclosed microcolonies of yeasts and hyphae with distinct biological properties from planktonic forms [8]. Although* C. albicans* remains the principle etiological agent, reports on the prevalence of non-*albicans Candida* (NAC) species associated with invasive candidiasis reflect the significance of NAC in clinical samples.* C. tropicalis*,* C. krusei*, and* C. glabrata* are the predominant NAC isolates from neonatal candidemia [9–12]. Concern on the shift towards NAC species arising from antifungal resistance and side effects of conventional treatments has led the search for potential bioactive components from plants [10, 13].

**Figure 1:**
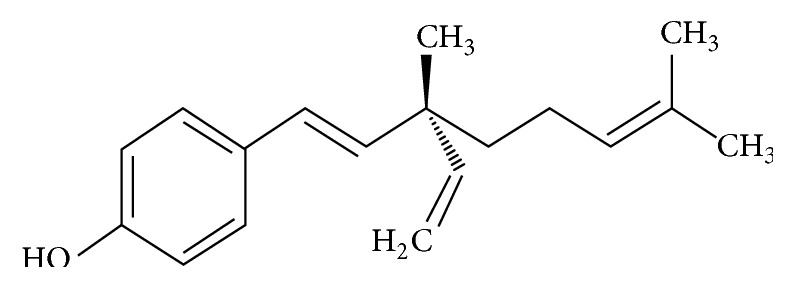
Chemical structure of bakuchiol.

Natural products from plants have been subjected for treatment because they are rich in a numerous variety of secondary metabolites with antimicrobial properties [14]. This has prompted the research interest on novel mechanism of action of plant-derived bioactive compounds for better therapeutic strategies. Bakuchiol (Figure 1) is one of active components of* Psoralea glandulosa* leaves, commonly used in folk medicine for the treatment of skin diseases caused by bacteria and fungi [15, 16]. It was also found in the seeds of* Psoralea corylifolia*. The seed oil has been used for the treatment of leucoderma, psoriasis, and leprosy [17], and the crude extract has been suggested as a remedy for bone fractures, osteomalacia, and osteoporosis [18]. Bakuchiol has shown diverse therapeutic properties including antibacterial and anticancer activities [19–21]. It is also reported to exhibit effectiveness towards pathophysiologic features of acne [22], suggesting its potential use in cosmetic formulation. Therefore, the present study aimed at investigating the antifungal properties of bakuchiol on oral-associated* Candida* species. The assessment of antifungal activity on* Candida* planktonic and biofilm was undertaken to characterise the mode of action of bakuchiol for the development of therapeutic agents specifically against candidal infections in the oral cavity.

## 2. Materials and Methods

### 2.1. *Candida* Strains and Bioactive Compound

*Candida* strains purchased from the American Type Culture Collection (ATCC),* C. albicans *ATCC 14053,* C. dubliniensis* ATCC MYA-2975,* C. glabrata* ATCC 90030,* C. krusei* ATCC 14243,* C. lusitaniae* ATCC 64125, and* C. tropicalis* ATCC 13803, were used in the study. Cells were stored at −70°C as glycerol stocks and propagated by streaking a loopful of cells onto yeast peptone dextrose (YPD) agar (15% w/v yeast extract, 31% w/v peptone, 31% w/v dextrose, 23% w/v agar) and incubated overnight at 37°C. Bakuchiol is one of major components of* Psoralea corylifolia* L. seed extract, identified by UV, IR, Mass, 1H, and 13C NMR spectra and melting point [23]. For this* in vitro* study, bakuchiol (purity: ≥95% HPLC) purchased from ChromaDex Inc. was dissolved in 1% v/v dimethyl sulfoxide (DMSO) (a stock concentration of 1000 *μ*g/mL) and stored at −20°C until use. Amphotericin B (Sigma-Aldrich; purity: ~80% HPLC) was used as a positive control in the experimental assays.

### 2.2. Culture Condition and Cell Inoculum

A single colony was inoculated into 10 mL YPD medium (20% w/v yeast extract, 40% w/v peptone, and 40% w/v dextrose) and grown overnight in an orbital shaker (150–180 rpm) at 30°C. Under this condition,* Candida* grows as budding yeast [24]. Cell cultures were harvested by centrifugation at 2000 ×g and washed with phosphate-buffered saline (PBS; 10 mM phosphate buffer, 2.7 mM potassium chloride, and 137 mM sodium chloride, pH 7.2). A standard inoculum was then adjusted to 1 × 10^6^ cells/mL (OD550 nm = 0.144).

### 2.3. Antifungal Susceptibility Testing

The minimum inhibitory concentration (MIC) assay was carried out by the standard broth microdilution method in YPD medium according to the Clinical and Laboratory Standards Institute (CLSI) reference method M27-A3 [25]. Inoculum of 1 × 10^3^ yeast cells/mL was added to each well of microtiter plates containing different concentrations of bakuchiol which ranged from 1.5 to 100 *μ*g/mL. The plates were incubated overnight at 37°C. The MIC endpoint was determined as the lowest concentration that caused a significant diminution (≥50% inhibition) of growth relative to the untreated groups [26]. Following this, the minimum fungicidal concentration (MFC) assay was carried out by spreading aliquots of 50 *μ*L from the well showing no visible growth on YPD agar plates. Absence of viable growth following 24 to 48 h incubation indicated the MFC of compound on the respective strains.

### 2.4. Growth Kinetics

To analyse the effect of bakuchiol on* Candida* growth, the yeast cells (1 × 10^3^ cells/mL) prepared in Section 2.2 were exposed to 0.5 × MIC of bakuchiol for 30 min. The treated cells were centrifuged, washed, and resuspended in PBS. These cells were then grown in YPD medium at 37°C for 18 h. At stipulated time intervals (3, 6, 9, and 12 h), the cell growth was measured at 550 nm. The growth of respective* Candida* species was distinguished by measuring the specific growth rate (*μ*) using the equation previously described [27]:

(1) $$\begin{array}{c} \mu =\frac{\left[\mathrm{In}N_{t}-\mathrm{In}N_{0}\right]}{t_{2}-t_{1}}, \end{array}$$

where *μ* is the average specific growth rate, *N*_*t*_ represented the number of cells at log phase, *N*_0_ represented the number of cells at zero time, *t*_2_ was the time taken to reach plateau, and *t*_1_ was the time when the cells entered the log phase. *μ* values were distinguished from the exponential phase between 6 h to 12 h, during which the cells appearing per unit time were proportional to the present population. The percent (%) inhibition in average specific growth rate following bakuchiol exposure was then calculated. The cell growth was further determined based on viable counts (CFU). After 12 h, 100 *μ*L from each well was aspirated and serially diluted tenfold in sterile distilled water. 100 *μ*L of each dilution was spread on YPD agar. Following 48 h incubation at 37°C, the CFU was enumerated.

### 2.5. Cell Surface Hydrophobicity

For this assay, the inoculum of 1 × 10^8^ yeast cells/mL (OD550 nm = 0.5) was prepared. The hydrophobicity of untreated and bakuchiol-treated planktonic cells was determined by the biphasic hydrocarbon/aqueous method according to Anil et al. [28]. Aliquots 5 mL of inoculum were centrifuged at 8000 ×g, and the solution was discarded. The pellets were briefly exposed to 0.5 × MIC of bakuchiol for 15 min. The treated cells were centrifuged, washed, and resuspended in PBS. 4 mL from each sample was transferred into glass tubes, and the absorbance (*A*_0_) was measured at 550 nm. Following this, 200 *μ*L of hexadecane (Sigma-Aldrich) was added, vortexed vigorously, and left at room temperature for 30 min to allow for cells separated into biphasic state. The absorbance of the lower hydrophilic layer (*A*_1_) was measured again. The CSH of each* Candida* was expressed as the percentage decrease in optical density of the aqueous phase of the test using the following formula:

(2) $$\begin{array}{c} \text{Change in }A550\left(\;\%\;\right)=\left[\;\frac{A_{0}-A_{1}}{A_{0}}\;\right]\times 100. \end{array}$$

### 2.6. Biofilm Formation

Using the cell inoculum prepared in Section 2.2, both single- and mixed-species biofilms of* Candida* were allowed to form on commercially available polystyrene, flat-bottom 96-well microtiter plates (Thermo Scientific Nunc) [29]. For mixed-species biofilms, the cell suspension was instead replaced with* C. albicans* and each of NAC species at a ratio 1 : 1. The surface of wells was coated with 50 *μ*L of clarified saliva and incubated for 90 min at 37°C. The saliva was then aspirated and 20 *μ*L of cell suspension of the respective single and mixed species was added into each well to form the single and mixed biofilms. After 60 min of adhesion phase, 100 *μ*L of YPD medium was added into each well, and the plates were incubated overnight in a rotary shaker at 37°C. After biofilm formation, the medium was aspirated, and the biofilms were gently washed with PBS to remove nonadherent cells. Biofilm biomass and the cellular metabolic activity were quantitatively measured using crystal violet staining and XTT reduction assay, respectively.

### 2.7. Biofilm Quantitation

#### 2.7.1. Crystal Violet Assay

*Candida* planktonic cells (Section 2.2) were exposed to 0.5 × MIC of bakuchiol for 30 min. Biofilms were then developed using these cells following the same procedure described in Section 2.6. After washing, the biofilms were fixed with ethanol and stained with 50 *μ*L of 0.1% w/v crystal violet solution for 15 min without agitation. The biofilms were washed three times and destained with 95% v/v ethanol, following which 75 *μ*L of the solution was transferred into new wells, and their absorbance was measured at 595 nm using a microtiter plate reader (SpectraMAX 340 Tunable Microplate Reader).

#### 2.7.2. XTT Reduction Assay

This assay was undertaken to examine the effect of bakuchiol on viability of* Candida* cells within the biofilms, which relies on the reduction of yellow tetrazolium salt XTT by dehydrogenase enzymes of metabolically active cells yielding an orange-coloured, water-soluble formazan [30]. Biofilms established from inoculum prepared in Section 2.2 were exposed to 0.5 × MIC of bakuchiol for 2 h. Afterwards, a total of 100 *μ*L XTT-menadione (10 : 1) solution, consisting of XTT sodium salt (Sigma-Aldrich) mixed with menadione (Sigma-Aldrich) solution, (1 mM in acetone; Sigma-Aldrich) was dispensed into each well. The plate was covered in aluminium foil and incubated in the dark for 2 h at 37°C. Following this, 75 *μ*L of the solution was transferred into new wells, and the amount of colorimetric change (a reflection of the metabolic activity of biofilm cells) was measured at 490 nm.

### 2.8. Statistical Analysis

Statistical analyses were performed using SPSS software (version 18.0). An independent *t*-test was used to compare the significant differences between controls (untreated) and bakuchiol-treated samples. A *P* value of < 0.05 was considered statistically significant.

## 3. Results

### 3.1. Antifungal Susceptibility of Planktonic Cells

The antifungal activity of bakuchiol against* Candida* species was tabulated in Table 1. The MIC and MFC endpoints were ranged from 12.5 to 100 *μ*g/mL.* C. albicans* and* C. dubliniensis* were found to be the most susceptible to bakuchiol, and* C. glabrata *was the least susceptible to bakuchiol.

### 3.2. Effect of Bakuchiol on Cell Growth

A significant difference in* Candida* cell growth was observed after 6 h, which indicates the exponential stage of* Candida* strains. Bakuchiol exhibited considerable growth inhibitory effect against most tested strains (Table 2). In detail, *μ* values of* C. albicans*,* C. dubliniensis*, and* C. lusitaniae* were markedly reduced by >50% compared with the untreated yeast cells (*P* < 0.05). On the other hand,* C. glabrata* and* C. tropicalis* were reduced to 38% and 35%, respectively. No significant differences were observed for* C. krusei* (11%). Based on CFU, the population of* C. albicans*,* C. dubliniensis*,* C. glabrata*,* C. krusei*, and* C. lusitaniae* were decreased by 1.2- to 1.5-fold change (*P* < 0.05) following exposure to bakuchiol. No significant differences were observed for* C. tropicalis* (*P* = 0.19).

### 3.3. Effect of Bakuchiol on Cell Surface Hydrophobicity

**Figure 2:**
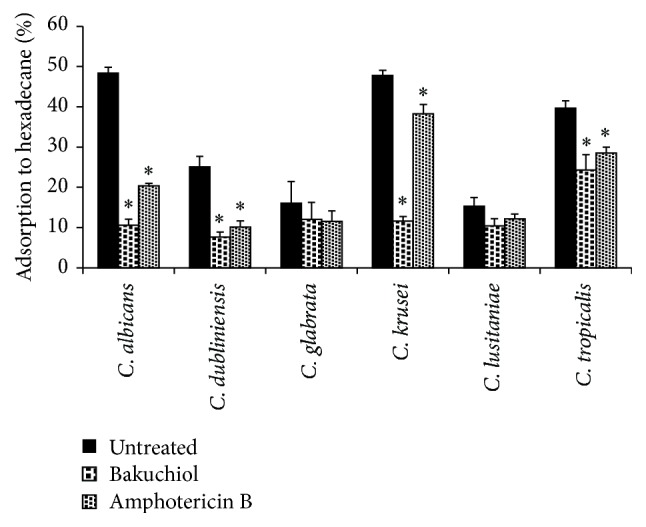
Cell surface hydrophobicity of* Candida* species following bakuchiol exposure. Data are represented as mean ± SD of three independent experiments performed in triplicate. Amphotericin B used as a positive control. Asterisk (*∗*) denotes the significant difference between treated samples and the untreated ones (one-way ANOVA; *P* < 0.05).

The effect of bakuchiol on cell surface hydrophobicity (CSH) of* Candida* species was measured based on the percentage of cell adsorption to hexadecane (Figure 2). The percentages of* C. albicans*,* C. krusei*, and* C. tropicalis* were significantly higher (*P* < 0.05) than those of the other* Candida* species. Findings show that the cell hydrophobicities were relatively compromised and decreased following brief exposure of* Candida* planktonic cells to 0.5 × MIC of bakuchiol. The CSH of* C. albicans*,* C. dubliniensis*,* C. krusei*, and* C. tropicalis* decreased within the range of 10% to 38% (*P* < 0.05). Bakuchiol, however, exhibited least effect on* C. glabrata* and* C. lusitaniae* when compared with the untreated yeast cells.

### 3.4. Effect of Bakuchiol on* Candida* Biofilm

**Figure 3:**
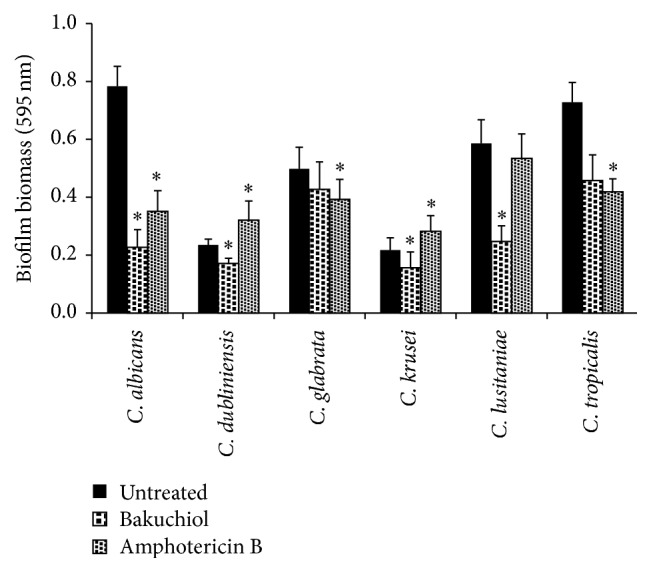
Absorbance values of crystal violet solutions obtained from* Candida* biofilm formation following bakuchiol exposure. Data were represented as mean ± SD of three independent experiments performed in triplicate. Amphotericin B used as a positive control. Asterisk (*∗*) denotes the significant difference between treated samples and the untreated ones (one-way ANOVA, ^*∗*^ *P* < 0.05).

*Candida* species were able to produce moderate-to-high degree of biomass after 24 h cultivated in a microtiter plate.* C. albicans* and* C. tropicalis* produced a dense biomass distinct from* C. dubliniensis*,* C. glabrata*,* C. krusei*, and* C. lusitaniae* (*P* < 0.05). Bakuchiol exposure showed significant reduction in most* Candida* biomass production, except for* C. glabrata* (*P* = 0.29) (Figure 3). Based on XTT assay, both* C. albicans* and* C. glabrata* exhibited the highest XTT metabolic activity compared to the others. In mixed biofilms, XTT activity of* C. albicans* and* C. glabrata* remains high (*P* < 0.01) when compared to the other mixed culture biofilms (Table 3). Following bakuchiol exposure (2 h), XTT activity in* C. albicans* and* C. tropicalis* was reduced (*P* < 0.05) relative to the untreated samples. No significant differences were observed on other single species biofilms. For mixed biofilms, only* C. albicans* and* C. dubliniensis* were markedly reduced by >50% (*P* < 0.05), followed by mixed* C. albicans* and* C. krusei* biofilm in response to bakuchiol.

## 4. Discussion

The crude extract and secondary metabolites derived from plants serve as important fields of research for new antifungal agents [31]. The antimicrobial properties of bakuchiol have been reported in previous studies [19]. The present study was designed to assess the antifungal activity of bakuchiol on* Candida* species, commonly associated with oral infections.* C. albicans* and* C. dubliniensis* were shown to be susceptible to bakuchiol. Bakuchiol demonstrated the inhibitory effect on* C. albicans*,* C. dubliniensis*, and* C. lusitaniae* with the specific growth rates (*μ* values) reduced by more than 50% compared to the untreated samples. The efficacy of bakuchiol was further elucidated by the inhibition of CFU of* Candida* cells and marked difference can be seen after 6 h inoculation.

*Candida* yeast-like species may adopt adhesive hydrophobic interactions to mediate adherence to different host surfaces [32, 33], which is one of many types of adhesion mechanism demonstrated by* Candida*. The present study examined the affinity of* Candida* planktonic cells for hydrophobic surfaces, that is, cell surface hydrophobicity based on the microbial adhesion to hydrocarbon (MATH) testing [34]. Previous studies claimed that CSH of planktonic cells positively correlates with biofilm formation [35]. Distinction in cell surface physicochemical properties and presence of carbohydrate moiety may influence the cell affinity for hydrophobic interface [33]. In this study, exposing* Candida* planktonic cells to bakuchiol (0.5 × MIC) has shown significant difference in the percentage of CSH, especially for* C. albicans* which were markedly reduced relative to the untreated samples. Bakuchiol could have made the cell surface area undergo transient changes and the impairment of cell hydrophobicity may lead to reduced adhesion to hydrophobic interfaces [32]. This assay demonstrated that bakuchiol exposure has a considerable effect towards the hydrophobic interactions of* Candida* cells. The reduction in CSH following amphotericin B exposure was also reported in previous study [28].

Cell adhesion and biofilm formation are the key areas for the antifungal treatment. The CSH assay has given insights to extend investigation on* Candida* biofilm formation. It is postulated that the impaired hydrophobic interactions may compromise the affinity of planktonic yeast cells to adhere to form biofilms. The crystal violet assay demonstrated that bakuchiol shows significant decreases in biofilm formation of* C. albicans*,* C. lusitaniae*, and* C. tropicalis*. The planktonic cells of* C. dubliniensis*,* C. glabrata*, and* C. krusei* were shown to be less susceptible to bakuchiol and slightly reduced the biomass production when compared to the untreated ones. The results show that bakuchiol may act as effective as amphotericin B in reducing biofilm formation.

The efficacy of bakuchiol on the established biofilms was further evaluated through XTT reduction assay. The metabolic activities of* C. albicans*,* C. glabrata*,* C. krusei*, and* C. tropicalis* were decreased following bakuchiol exposure. No significant differences were observed on* C. dubliniensis* and* C. lusitaniae*. This could possibly be due to their lower metabolic activity which may result in reduced response to the treatment. Meanwhile, for mixed-species biofilms, the metabolic rates are varying according to the sensitivity of different* Candida* species within biofilms.* C. albicans* remains the principle species in biofilm formation due to germ tubes and hyphae formation. The hyphae-associated adhesins such as agglutinin-like sequence (ALS) and hyphal wall protein (HWP1) are crucial for adhesion [36, 37]. It is reported that* C. albicans* had a positive impact on certain NAC species in biofilm formation compared to when growing alone [38]. The NAC species may benefit from the interaction with* C. albicans* in mixed biofilms which further increased the cell metabolic activities and were inherently resistant to antifungal treatment. In the present study, the coculture of* C. krusei* and* C. albicans* was not highly proficient in forming dense biofilms. The cell number in mixed biofilm was also previously shown to be reduced when varying concentrations of* C. krusei* were cocultured with a constant concentration of* C. albicans* [36]. Johansson Wächtler et al. [37] reported that, unlike* C. albicans*,* C. krusei* was unable to utilize salivary statherin and mimicking molecules as functional adhesion molecules on salivary pellicles and epithelial cells. This possibly explains the less dense biofilm formed between* C. krusei* and* C. albicans*. Bakuchiol treatment, however, markedly reduced the metabolic activities of* C. albicans* cocultured with* C. dubliniensis* and* C. tropicalis*. The high susceptibility of* C. albicans* to bakuchiol may have influenced the mixed species in biofilms. The coculture of* C. albicans* and* C. glabrata* exhibited more resistance towards bakuchiol. This may indicate synergistic relationship between the two species towards antifungal resistance [39].

## 5. Conclusion

Bakuchiol exhibited antifungal activity against planktonic and biofilm forms of* Candida* species. Findings show that bakuchiol inhibited the planktonic growth and reduced the adhesive capacity of* Candida*.* C. albicans* and the NAC species, except for* C. glabrata*, have been shown to be susceptible to bakuchiol. Antifungal properties of bakuchiol in the present study could give insights for the development of a new therapeutic agent against the treatment of* Candida*-associated infections.

## Figures and Tables

**Table 1:** Antifungal activity of bakuchiol against *Candida *species.

	Antifungal susceptibility test^a^ (*µ*g/mL)					
	1	2	3	4	5	6
Bakuchiol						
MIC	25	12.5	>100	50	50	50
MFC	50	25	>100	50	100	100
Amphotericin B						
MIC	1.95	1.95	1.95	1.95	7.81	1.95
MFC	3.91	1.95	3.91	1.95	7.81	3.91

^a^(1) *C. albicans *ATCC 14053, (2) *C. dubliniensis* ATCC MYA-2975, (3) *C. glabrata* ATCC 90030, (4) *C. krusei* ATCC 14243, (5) *C. lusitaniae* ATCC 64125, and (6) *C. tropicalis* ATCC 13803.

**Table 2:** Changes in the specific growth rates (*µ*) and the viable counts (CFU) of *Candida *species following bakuchiol exposure. The percentage denotes the inhibition of treated samples compared to the untreated ones.

Microorganism	Treatment^a^	*µ* (%)	Log_10_ CFU/mL (%)
*C. albicans* ATCC 14053	Untreated	1.73 ± 0.08	10.04 ± 0.03
	Bakuchiol	0.70 ± 0.07 (59.5)^*∗*^	7.24 ± 0.08 (27.9)^*∗*^
	Amphotericin B	0.49 ± 0.10 (71.7)^*∗*^	6.64 ± 0.05 (33.9)^*∗*^

*C. dubliniensis* ATCC MYA-2975	Untreated	0.98 ± 0.14	8.32 ± 0.28
	Bakuchiol	0.45 ± 0.24 (54.1)^*∗*^	6.87 ± 0.42 (17.4)^*∗*^
	Amphotericin B	0.74 ± 0.19 (24.5)^*∗*^	7.36 ± 0.09 (11.5)^*∗*^

*C. glabrata* ATCC 90030	Untreated	1.80 ± 0.06	10.13 ± 0.08
	Bakuchiol	1.11 ± 0.03 (38.3)^*∗*^	8.34 ± 0.07 (17.7)^*∗*^
	Amphotericin B	1.47 ± 0.13 (18.3)^*∗*^	9.20 ± 0.07 (9.2)^*∗*^

*C. krusei* ATCC 14243	Untreated	0.82 ± 0.20	8.63 ± 0.45
	Bakuchiol	0.73 ± 0.15 (11)	5.72 ± 0.35 (33.7)^*∗*^
	Amphotericin B	0.55 ± 0.12 (32.9)^*∗*^	6.32 ± 0.21 (26.8)^*∗*^

*C. lusitaniae* ATCC 64125	Untreated	1.09 ± 0.15	8.95 ± 0.36
	Bakuchiol	0.42 ± 0.10 (61.5)^*∗*^	6.90 ± 0.16 (22.9)^*∗*^
	Amphotericin B	0.98 ± 0.24 (10.1)	8.69 ± 0.50 (2.9)

*C. tropicalis* ATCC 13803	Untreated	1.57 ± 0.14	9.80 ± 0.43
	Bakuchiol	1.02 ± 0.18 (35)^*∗*^	9.30 ± 0.23 (5.5)
	Amphotericin B	0.91 ± 0.12 (42)^*∗*^	8.63 ± 0.08 (11.9)^*∗*^

^a^Test concentration was prepared at 0.5 × MIC. ^*∗*^ *P* < 0.05 compared to the untreated samples.

**Table 3:** The metabolic activity of single and mixed biofilms when exposed to bakuchiol was measured. Values represent absorbance using XTT reduction assay.

Biofilms	Untreated	Bakuchiol	Amphotericin B
Single species			
*C. albicans*	1.042 ± 0.049^†^	0.417 ± 0.085^*α*^	0.266 ± 0.046^*α*,*β*^
*C. dubliniensis*	0.279 ± 0.016	0.274 ± 0.029	0.174 ± 0.026^*α*,*β*^
*C. glabrata*	0.723 ± 0.040^†^	0.896 ± 0.096	0.353 ± 0.061^*α*,*β*^
*C. krusei*	0.467 ± 0.048	0.400 ± 0.085	0.228 ± 0.079^*α*,*β*^
*C. lusitaniae*	0.192 ± 0.016	0.182 ± 0.017	0.242 ± 0.030^*α*,*β*^
*C. tropicalis*	0.351 ± 0.037	0.243 ± 0.032^*α*^	0.376 ± 0.056^*β*^

Mixed species			
*C. albicans*			
+ *C. dubliniensis*	0.435 ± 0.024	0.205 ± 0.015^*α*^	0.201 ± 0.023^*α*^
*+ C. glabrata*	1.023 ± 0.054^†^	0.962 ± 0.104	0.527 ± 0.036^*α*,*β*^
*+ C. krusei*	0.444 ± 0.014	0.315 ± 0.018^*α*^	0.330 ± 0.032^*β*^
*+ C. lusitaniae*	0.292 ± 0.013	0.246 ± 0.014	0.232 ± 0.025
*+ C. tropicalis*	0.289 ± 0.019	0.210 ± 0.025	0.198 ± 0.015^*β*^

^†^ *P* < 0.01 compared to others in their respective groups; single and mixed species biofilms.

^*α*^ *P* < 0.05 compared to the untreated samples.

^*β*^ *P* < 0.05 compared to bakuchiol-treated samples.
